# A qualitative study on the current status and problems of pharmacists in home healthcare from the viewpoint of care managers in medically underpopulated areas in Japan

**DOI:** 10.1186/s40780-024-00395-1

**Published:** 2024-11-08

**Authors:** Yuji Nakagawa, Hideo Kato, Takuya Iwamoto

**Affiliations:** 1 Department of Clinical Pharmaceutics, Faculty of Medicine, Mie University Graduate School of Medicine, 174-2, Edobashi, Tsu, Mie, 514-8507 Japan; 2 Department of Pharmacy, Mie University Hospital, 174-2, Edobashi, Tsu, Mie, 514-8507 Japan

**Keywords:** Home healthcare, Medically underpopulated area, Community pharmacist, Qualitative research, Japan

## Abstract

**Background:**

Unlike in urban areas, community healthcare in medically underpopulated areas in Japan is constantly challenged because of the uncertainty in effectively using the limited resources. However, no study has focused on human resources or identified the actual state of pharmacists’ support in the community. Therefore, our study identified the actual status and problems of pharmacists involved in home medical care in medically underpopulated areas and discussed the roles required of pharmacists and specific methods of support.

**Methods:**

The content of semi-structured interviews with care managers involved in home healthcare in Misugi town, Tsu City, between November 10, 2023 and March 13, 2024, was analyzed qualitatively using the grounded theory approach.

**Results:**

Five care managers participated in the study. Semi-structured interviews on the actual situation and challenges faced by pharmacists indicated that the following roles were required for pharmacists: as in other regions, it was observed that elderly people with dementia and those living alone managed their medicines, adjusted leftover medicines, collaborated with other professions, bridged with physicians, checked medication status through frequent visits, and adhered to internal medication regimens. Issues related to the characteristics of depopulated areas were identified including human resources, limitations of healthcare resources, economic burden, limits on the number of visits by pharmacists. As a characteristic of communities with no pharmacies and only in-hospital prescribing, pharmacists were expected by care managers to manage the problems caused by in-hospital prescribing.

**Conclusions:**

Our findings suggest that pharmacists should ensure the number of visits and collaborate with attending physicians, visiting nurses, and care managers to conduct drug management for patients with dementia and older adults living alone. Community healthcare specialists and those involved in the healthcare planning system can also utilize these findings while planning home healthcare to those who live in medically underpopulated areas in Japan.

## Background

According to a survey conducted by the National Institute of Population and Social Security Research, the aging rate in Japan is expected to reach 38.4% by 2060 [1]. Consequently, the medical care system is shifting from inpatient care at medical institutions to home healthcare because the number of chronically ill patients requiring long-term care and the older adults requiring nursing care will increase [2]. However, all patients are not in an environment where they can receive sufficient medical intervention in hospitals or at home. In underpopulated areas, such as mountain villages and remote islands, the lack of human and natural resources has prevented the development of health and medical welfare services and other systems [3] because these areas often lack geographical features and available transportation options. In order to promote the involvement of pharmacists in home healthcare based on comprehensive community care, it may be essential to collaborate with care managers who play an important role as a bridge between medical professionals and care workers in team healthcare in the community [4].

Misugi Town, Tsu City, Mie Prefecture, is located in the southwestern part of Tsu City and occupies approximately 30% of the total area of Tsu City. Misugi has a population of 3,639, with 62.6% of the population aged 65 years or older (the national average is 29.1%) [5]. The aging population rate is increasing owing to population decline and the outflow of young people. Misugi is designated as a depopulated area by the Law on Special Measures for the Promotion of Self-Support of Depopulated Areas [6]. There are no pharmacies in the study area and no pharmacy pharmacists are employed in the study area. Looking at the number of pharmacists per 100,000 population by prefecture, the national average is 202.6, while the number of pharmacists in Mie Prefecture is 3,550, and the number of pharmacists per 100,000 population in Mie Prefecture is 200.5. In particular, the number of pharmacy pharmacists per 100,000 population in Mie Prefecture was 129.4, which is below the national average of 149.8.

Unlike in urban areas, community healthcare in medically underpopulated areas faces the issue of effective utlilization of limited resources. Therefore, it is necessary to understand the status of implementation of home support by medical and nursing care staff. However, no survey has focused on human resources or clarified the actual status of pharmacists’ support in medically underpopulated areas. Therefore, this study examined the actual status and problems of pharmacists involved in home healthcare in medically underpopulated areas and discussed the roles required by pharmacists and specific methods of support from the care manager’s perspective.

## Methods

### Research participants

Care managers involved in home healthcare in Misugi Town, Tsu City, Mie Prefecture were included in this study. The number of participants is not defined a priori, as the GTA-based analysis method is used. Data collection and analysis will be completed when theoretical saturation is reached. The approximate number of candidates should be approximately 5–10 participants. Participation criteria are care managers who are involved in home healthcare in Misugi Town and who have given consent for the interview survey. Exclusion criteria are those who do not wish to participate in the interview survey and those who are deemed inappropriate for inclusion in the study by the principal investigator/associate investigator.

### Data collection

From November 10, 2023 to March 13, 2024, one-on-one semi-structured interviews were conducted with each care manager according to the questions listed in Table 1. Interviewees were asked about their involvement with pharmacists in home care in depopulated areas, multidisciplinary collaboration, communication with pharmacists, and expectations of pharmacists. There was no limit to the length of the interviews. Interviews were recorded with an integrated circuit recorder to create a verbatim transcript and analyzed qualitatively using the grounded theory approach (GTA) [7–9]. The termination of the analysis in GTA was based on theoretical saturation; when no new categories were found in an interview with a certain participant, it was determined that theoretical saturation had been reached and no additional surveys were conducted [7–9].

**Table 1:** Semi-structured interview questions

1.	Have you ever been involved with a pharmacist on the problem of a patient receiving home healthcare in an underpopulated area? What was the content? Were you satisfied with the results regarding its content? If not, what was lacking?
2.	Have you ever had a problem with a patient receiving home healthcare in an underpopulated area that you thought required the intervention of a pharmacist?
3.	Have you had any problems with drug treatment of patients receiving home healthcare in underpopulated areas? With whom would you discuss these problems?
4.	Please tell us about a good experience you had with a pharmacist, including a patient problem.
5.	In your interactions with pharmacists, what do you find to be a problem, including patient problems?
6.	Do you collaborate with other professions regarding patients receiving home healthcare in underpopulated areas? If yes, what kind of collaboration do you have? If not, have there been situations in which you have not been able to do so?
7.	Did you experience any difficulties in communicating with pharmacists? What were the specific problems? How do you think you should handle them?
8.	What are your expectations for pharmacists in home healthcare in depopulated areas?
9.	Based on your experience, do you think what is necessary to achieve home healthcare in ideal depopulated areas?

### Data analysis

Audio recordings of the interviews were converted into textual data and quantitatively analyzed using Strauss and Corbin’s GTA [9–12]. The data were sectioned based on contextual content. Based on the sectioned data, the properties (characteristics or components of objects, events, and actions) and dimensions (positions of data in terms of properties) were extracted as subordinate concepts. Labels (concept names with a slightly higher level of abstraction) were then assigned to appropriately represent the intercepts [10]. In the GTA, the use of properties and dimensions provides a logical basis for interpretation [9]. In other words, the transcripts made from the interview recordings were intercepted for each semantic unit, and then the properties and dimensions in the intercepts were extracted and the names of the labels were determined. The created labels were grouped into categories, taking into account the properties and dimensions, and then a category association diagram was created, considering the relationship between the categories involved in each situation. Based on this, a conceptual diagram was created to grasp the overarching concept, and the phenomenon was theorised. The labels were classified to categories which are more abstraction level concepts. One researcher (YN) created the properties, dimensions, labels, and categories, and another researcher (HK) checked them. The obtained data were analyzed individually, and when no new concepts could be created, the data were considered theoretically saturated [7–9].

### Ethical considerations

This study was conducted in compliance with the Ethical Guidelines for Medical Research Involving Human Subjects and was based on a research plan approved by the Ethical Review Committee for Medical Research at Mie University Hospital (No. H2023-188). Participants were informed about the purpose and methods of the study, their free will to participate, the possibility of withdrawing from participation at any time, avoidance of disadvantages, anonymization, and protection of personal information using an explanatory document. After confirming that they were completely convinced, they signed a consent document. Those who refused to participate in the interviews were excluded.

## Results

### Overview of the interviews

There are only five care managers in the study area who were interviewed for this study. In addition, the five care managers belong to one office. In addition, there is only one office in the study area. Theoretical saturation was achieved by five interviewees because no new concepts were extracted. The backgrounds of all the care managers had a caregiver background and no medical qualifications. Interviewee 1 was interviewed for 25 min, 2 for 29 min, 3 for 20 min, 4 for 15 min, and 5 for 15 min. The interviewees’ attributes are listed in Table 2. The interviewees included one male and four females. The median career duration as a care manager was 11 years. The following three situations were extracted: (1) the actual situation of home healthcare in medically underpopulated areas and pharmacists; (2) problems for pharmacists in home healthcare in medically underpopulated areas; and (3) Roles required of pharmacists in home healthcare in medically underpopulated areas. The results of the analysis for each scenario are described below.

**Table 2:** Background of interviewees

Participant	Sex	Profession	Career	Frequency of visits with pharmacist
1	Female	Care manager	11 years	Frequent visits when needed
2	Female	Care manager	23 years	Once a year (At the Community Care Conference)
3	Female	Care manager	10 years	Once every six months
4	Female	Care manager	20 years	Once every six months
5	Male	Care manager	10 years	Almost none

### Actual situation of home healthcare in medically underpopulated areas and pharmacists

**Fig. 1:**
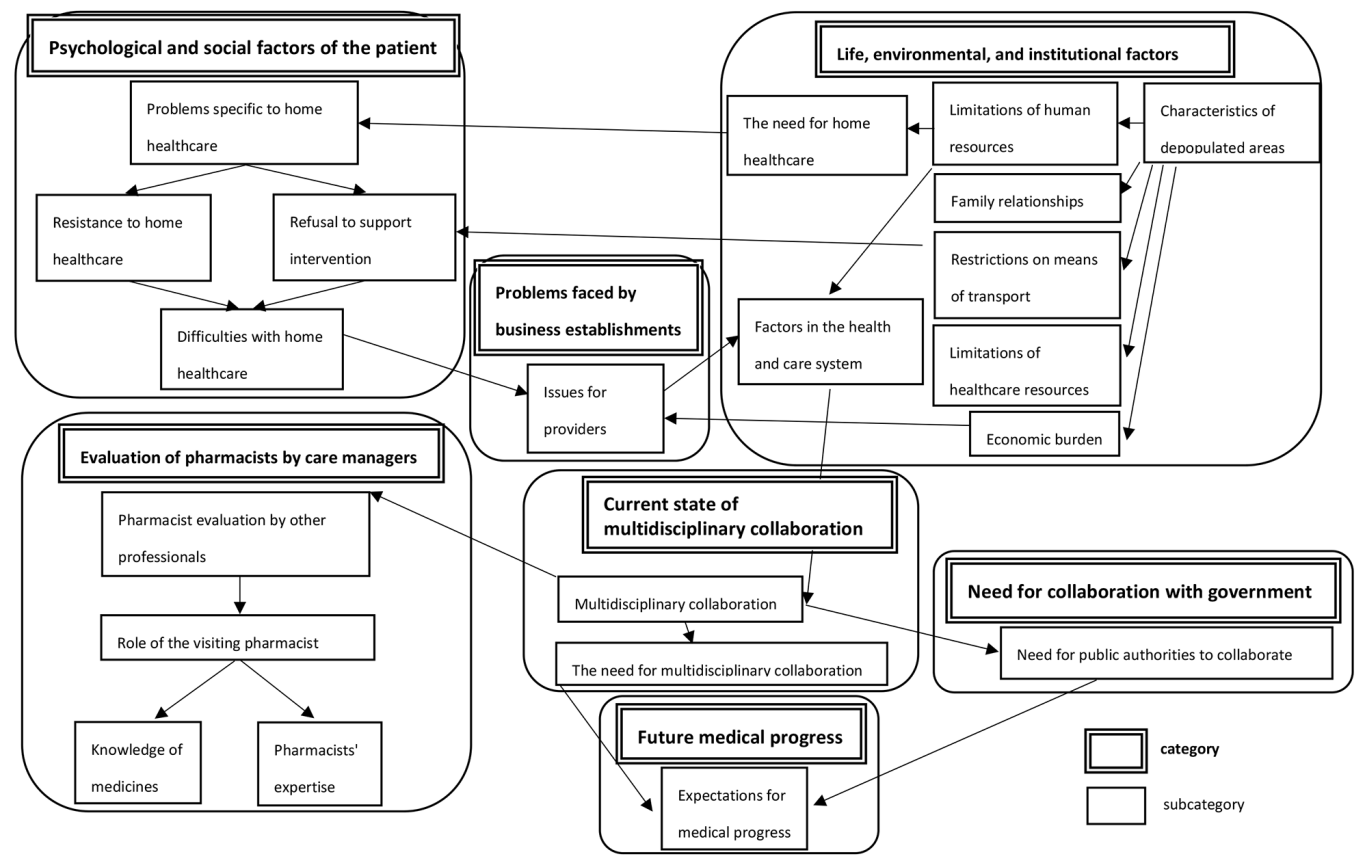
Category correlations on the actual situation of home healthcare and pharmacists in medically underpopulated areas

Seven factors were examined as the current state of home healthcare in medically underpopulated areas and the actual situation of pharmacists: “psychological and social factors of the patient”; “life, environment, and institutional factors”; “current state of multidisciplinary collaboration”; “evaluation of pharmacists by care managers”; “problems faced by business establishments”; “need for collaboration with government administration”; and “future medical progress” (Fig. 1). The main labels for each factor are also shown to Table 3. These labels are component of the subcategory. Regarding the category of “psychological and social factors of the patient”, the item of “problem specific to home healthcare” is that many patients wish to receive end-of-life care at home, and the unwillingness to involve others leads to “resistance to home care”. Resistance was defined as the minimal use of welfare services in the lives of patients with low levels of medical dependency, refusal of intervention by non-caregivers from a financial point of view, and refusal to accompany patients to outpatient clinics. Refusal to accompany patients to outpatient clinics was revealed as “refusal to support intervention” and was caused in part by “problems specific to home healthcare” (Fig. 1). Regarding “living, environmental and institutional factors”, the “limitation of human resource” was also identified. In terms of relationships with family members, there was a lack of family involvement, including a lack of understanding by families of elderly households with dementia.

**Table 3:** Labels and categories of actual situation of home healthcare and pharmacists in medically underpopulated areas

Main labels	Subcategory	Category
The desire to end up at home	Problems specific to home healthcare	Psychological and social factors of the patient
Living with low levels of medical dependency, Minimal use of welfare services, Refusal to intervene from non-caregivers due to financial considerations.	Resistance to home healthcare	
Refusal to accompany clients to outpatient clinics	Refusal to support intervention	
Difficulties in home care for users requiring nursing care 3 and above	Difficulties with home healthcare	
Areas where people are treated at home to the limit, Old age and few medical resources, Elderly care and limited medical resources, The lives of users who can no longer drive, Inability to obtain out-of-hospital prescriptions in close proximity, Inconvenience of not having a dispensing pharmacy	Characteristics of depopulated areas	Life, environmental, and institutional factors
Limitations of health care resources in the Misugi area	Limitations of healthcare resources	
Limitations of human resources of home care nurses	Limitations of human resources	
Relationships with families of older people with dementia, Lack of understanding of families of elderly household members with dementia, Lack of engagement with families, How to deal with family members,	Family relationships	
The need for home visiting services in depopulated areas, Transition to home visiting services	The need for home healthcare	
Patients unable to pick up their medicines on their own	Restrictions on means of transport	
Economic burden due to health services, Restriction of use of services due to financial burden	Economic burden	
Limits on the number of visits by pharmacists	Factors in the health and care system	
Ease of multidisciplinary collaboration, Good system of collaboration with multiple professions, Practice of multidisciplinary collaboration with visiting nurses, Confirmation of medication status by multidisciplinary staff, Experience of multidisciplinary cooperation	Multidisciplinary collaboration	Current state of multidisciplinary collaboration
The need for multidisciplinary help	The need for multidisciplinary collaboration	
Economic burden on operators, Limited establishments and day services, Withdrawal of establishments due to declining numbers of users	Issues for providers	Problems faced by business establishments
Good relationship with pharmacists, Checking for residual medication and careful medication guidance, Appreciation of drug distribution and medication guidance through visits Satisfaction reported, Satisfaction with the relationship with the pharmacist, Smooth communication	Pharmacist evaluation by other professionals	Evaluation of pharmacists by care managers
Medicines management and liaison with the attending physician, Residual medication reconciliation, On-site drug management for those unable to manage their medicines, Addressing medication errors, Intervention by pharmacists to identify problems	Role of the visiting pharmacist	
Current lack of understanding of medicines, Difficulties in answering questions about medicines, Difficulty in understanding terminology, Slippage of questions about medicines	Knowledge of medicines	
Checking of oral medication status and suggestions for reducing medication, Bridging the gap with the doctor, Good acceptance of explanations from doctors and pharmacists	Pharmacists’ expertise	
Resignation to inadequate administrative response, The need for support from the public administration	Need for public authorities to collaborate	Need for collaboration with government
Expectations for medical progress	Expectations for medical progress	Future medical progress

To solve the problems of the medical and long-term care systems, the current state of good collaboration among multiple professionals was observed. “The need for multidisciplinary collaboration” and support was well understood. Therefore, care managers were highly aware of “the need for multidisciplinary collaboration”, such as checking the status of medications by multidisciplinary professionals.

The subcategory of “Pharmacists evaluation by other professionals” shows that other professionals seem to have good relationships with pharmacists from the viewpoint of care manager. Care managers evaluated the careful attention of pharmacists to the patient’s needs, including checking leftover drugs, guiding medication carefully, and distributing drugs during patient visits. Pharmacists frequently provide patient information to care managers. As a result, care managers understood “the role of visiting pharmacists”.

In addition, as part of “multidisciplinary collaboration”, at present, there is a sense of resignation regarding the inadequate response of public administration. Among “the needs for multidisciplinary collaboration” and collaboration with public authorities, there was a desire to expect medical progress in the future.

### Problems of pharmacists in home healthcare in medically underpopulated areas

**Fig. 2:**
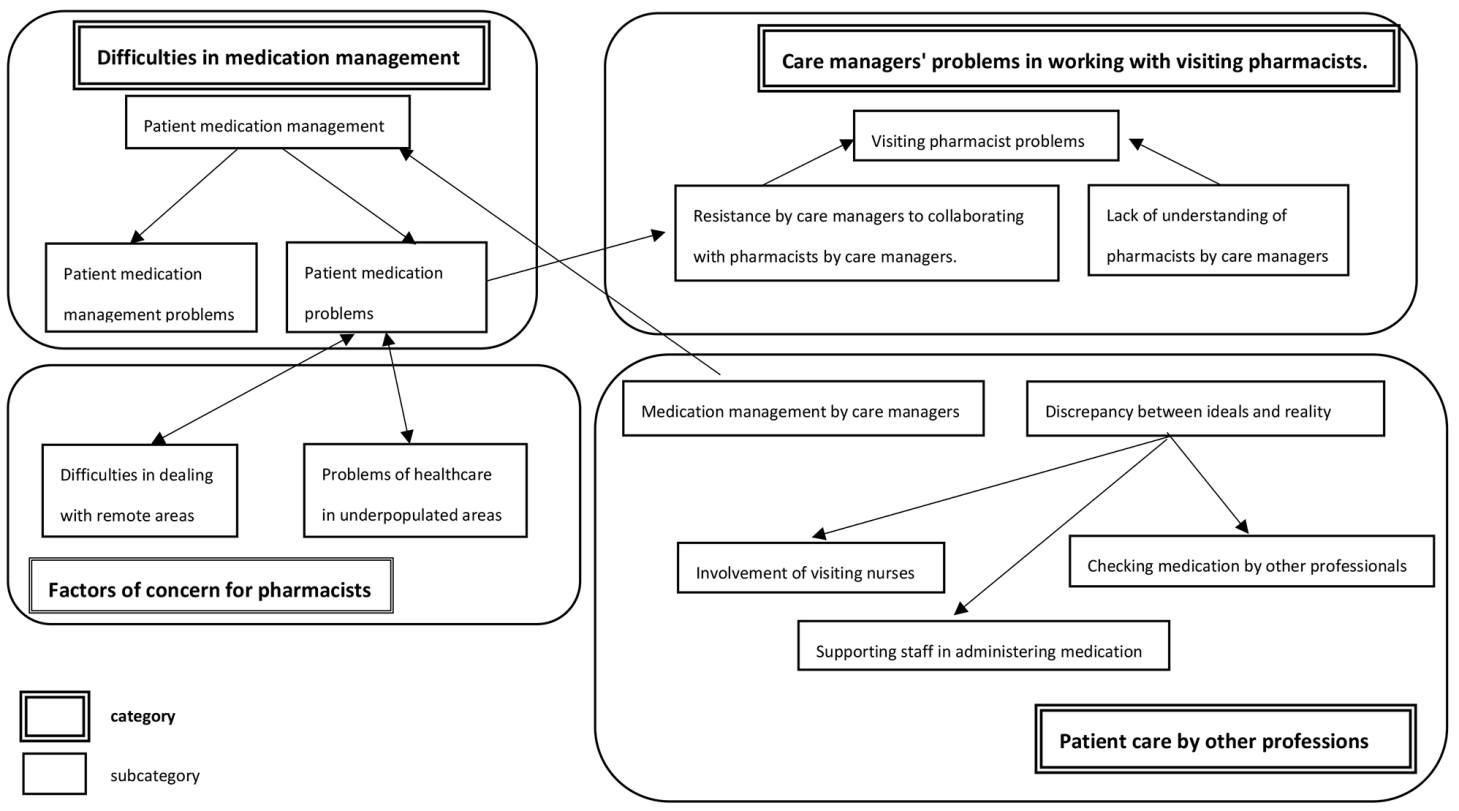
Category correlations on the problems of home healthcare and pharmacists in medically underpopulated areas

Care managers examined four factors as problems for pharmacists in home care in medically underpopulated areas: “difficulties in medication management”, “problems for visiting pharmacists”, “patient care by other professionals”, and “factors of concern for pharmacists” (Fig. 2). The main labels for each factor are also shown to Table 4. These labels are component of the subcategory. Intervention for patients with dementia is a key problem in the management of their medication. This was influenced by the absence of family members to assist in the management of medicines, resulting in concerns and anxiety about the medication situation and non-compliance. Moreover, there was a perception of resignation regarding medication management among patients with dementia living alone. This results in noncompliance, leading to the discovery of large quantities of leftover drugs. There are many problems with medication management, especially, patients’ leftover drugs, which can represent a significant problem in stabilising medication compliance. Therefore, visiting pharmacists need to check medication compliance through frequent visits as part of “patient’s medication management”.

**Table 4:** Labels and categories of problems for pharmacists in home care in medically underserved areas

Main labels	Subcategory	Category
Medication management and guidance on medication, Medication management using a medication calendar, Checking medication status through frequent visits	Patient medication management	Difficulties in medication management
Medication management of patients with dementia living alone, Interventions for the management of medication in patients with dementia	Patient medication management problems	
Absence of family members to support the management of medication, Concerns about the medication situation, Resignation to poor compliance, Problems with medication in patients with dementia, Difficulties in managing medication in elderly dementia patients, Giving up on medication for patients with dementia who live alone, Discovery of large quantities of leftover medication, Problems with how medicines are managed, Challenges in stabilising medication compliance	Patient medication problems	
Lack of confidence due to lack of knowledge of medicines	Resistance by care managers to collaborating with pharmacists by care managers.	Internal Problems for visiting pharmacists
Lack of engagement with pharmacists	Lack of understanding of pharmacists by care managers	
Past failure to get pharmacists to visit, Resignation to the lack of feasibility of pharmacist visits	Visiting pharmacist problems	
Medication management by visiting nurses, Dependence on visiting nurses, Smooth cooperation with home care nurses, Consultation with visiting nurses on medication management	Involvement of visiting nurses	Patient care by other professions
Unavoidable accompaniment to medical examinations	Supporting staff in administering medication	
Prompting of medication by care managers	Medication management by care managers	
Medication administration by caregivers, Checking medication by caregivers and nurses	Checking medication by other professionals	
Difficulties in visiting the backcountry, Difficulties in dealing with the backcountry	Difficulties in dealing with remote areas	Factors of concern for pharmacists
Problems that cannot be solved due to unprofitability, Few services in depopulated areas, Areas where home visits are not possible	Problems of healthcare in underpopulated areas	

The problems faced by visiting pharmacists included resistance to collaboration and a lack of understanding. This partly arose from the care managers’ lack of confidence due to their lack of knowledge about medicines and engagement with pharmacists. In some cases, problems with visiting pharmacists have been linked to their lack of feasibility of visiting pharmacists.

In terms of the relationship with visiting nurses, patients with a high level of medical dependency were actively administered medication by visiting nurses, indicating a dependence on visiting nurses. Care managers believed that they ensured smooth collaboration with visiting nurses and that they often consulted visiting nurses about medication management. In addition, as a support staff response to medication management, care managers communicated the status of internal medication to the primary care physician, checked the medication, and encouraged the patient to take the medication. In many cases, medication management was carried out by caregivers and nurses as a way for other professionals to check medication.

A problem that could not be solved for pharmacists was the distance problem for home visits in underpopulated areas. Owing to the lack of services in depopulated areas, there is a concern that patients cannot receive their scheduled medical care.

### Roles required of pharmacists in home healthcare in medically underpopulated areas

**Fig. 3:**
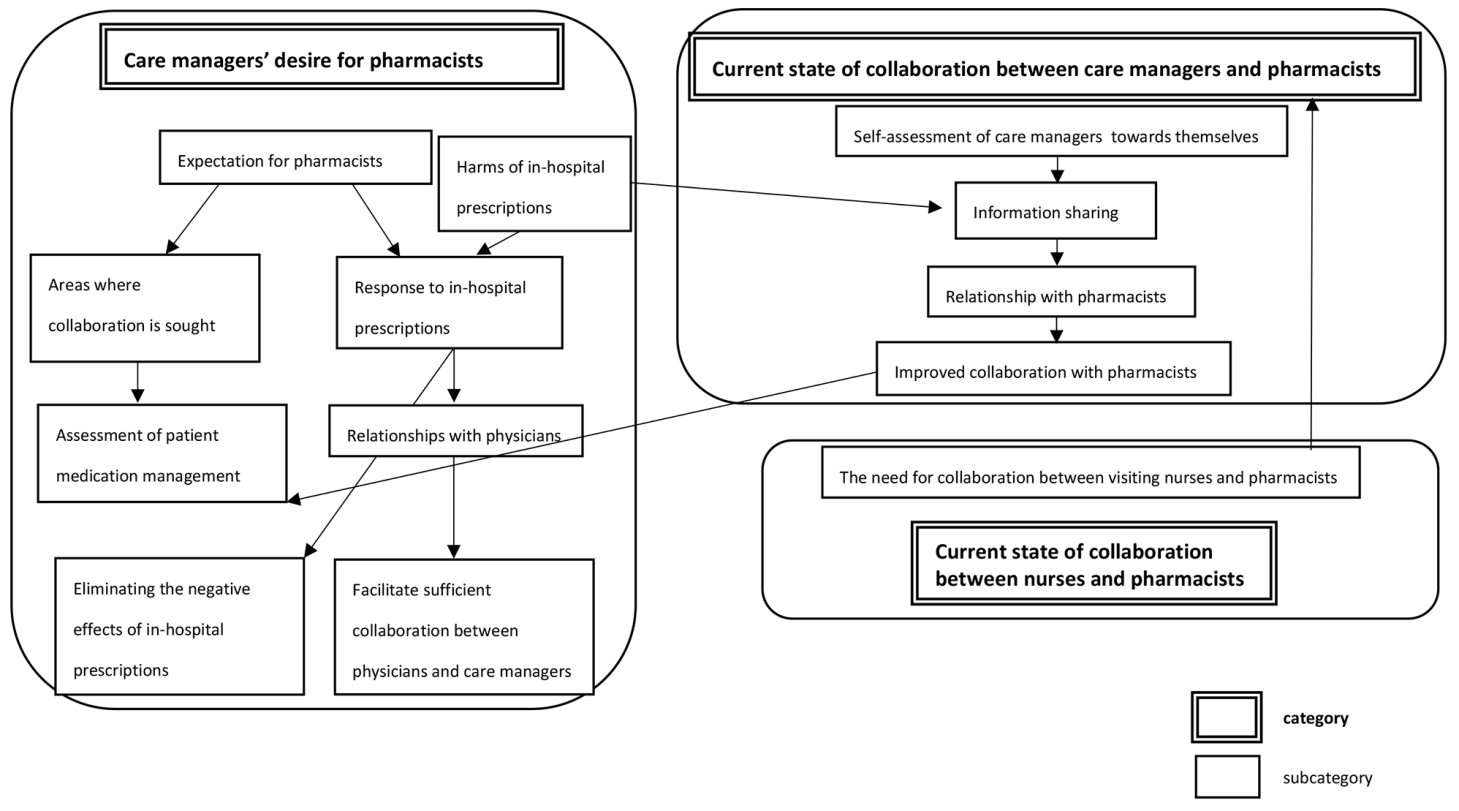
Category correlations on the roles required for pharmacists in home healthcare in medically underpopulated areas

Care managers examined three factors as the roles required of pharmacists in home care in medically underpopulated areas: “Care managers’ problems in working with visiting pharmacists” and “current state of collaboration between care managers and pharmacists” and “current state of collaboration between nurses and pharmacists” (Fig. 3). Among the “Care managers’ problems in working with visiting pharmacists “, improvements in medication management through pharmacists’ interventions were mentioned. In addition, the desire for more frequent and distant visits by pharmacists was mentioned. And it was extracted that care managers’ requests to pharmacists stemmed from their “expectations of pharmacists”, particularly for assessment of “patient medication management” and response to in-hospital prescriptions.

In areas where collaboration is expected, an assessment of “patient medication management”, such as monitoring the medication status of their medicines, is desired. Moreover, there was a desire to respond to in-hospital prescriptions and collaborate with primary physicians. In “the current state of collaboration between care managers and pharmacists”, low self-evaluation of care managers has been observed, which may lead to “insufficient collaboration with the attending physician” and may be a barrier to eliminating the negative effects of in-hospital prescriptions. Regarding “the current state of collaboration between nurses and pharmacists”, some expressed the desire for further collaboration between visiting nurses and pharmacists.

## Discussion

This study qualitatively analyzed the current situation of home healthcare from the perspective of care managers involved in home healthcare in medically underpopulated areas and evaluated the actual situation and problems faced by pharmacists in home healthcare and the required roles of pharmacists. In considering the direction of home healthcare as a concrete example of community healthcare, the quantity, quality and issues of required services will differ greatly depending on whether the target is a large city or a depopulated area. The home healthcare in large cities with large populations and sufficient medical resources has become well known as a system in which patients have a visiting pharmacist and can receive healthcare comparable to that provided in hospitals. In depopulated areas with small populations and limited medical resources, however, it is not always possible to achieve the same results as in large cities, even if by successful example of such areas is followed. In depopulated areas such as mountain villages and remote islands, the population is generally ageing and demands for medical and long-term care services are increasing, but due to a lack of personnel and resources, systems such as health and medical welfare services have not been developed [13].

Our study also reveals that underpopulated areas have limited medical and human resources. Serious problems such as financial burden on providers and the withdrawal of providers due to a decrease in the number of patients implementing home healthcare were also found. Additionally, many older adults are forced to live alone or with older caregivers, leading to reduced relationships with their families. And it was also clear that pharmacists’ visits were less frequent, as it was not easy for pharmacists to visit in the remote, underpopulated areas. This clearly shows that the characteristics of depopulated areas are widely influential (Fig. 1). A previous study reported the specific characteristics of drug treatment in patients undergoing home healthcare: visits to multiple hospitals, risk of increased leftover drugs, drug interactions and self-judged overdose, patients’ distrust of drugs, anxiety about taking drugs, awe or reticence towards physicians, and difficulty disclosing leftover drugs [14–16]. These characteristics appear to be similar to those of patients receiving home care in medically underpopulated areas.

Collaboration between physicians and pharmacists has been reported to be promoted in outpatient settings, and the information provided by pharmacists helps physicians make better decisions to review prescriptions, leading to more appropriate drug treatment [17]. Some cases have been reported in which pharmacists’ involvement in drug treatment and adverse drug reaction measurements was effective in multidisciplinary collaboration in the introduction of home care rehabilitation [18]. In addition, other professions expect pharmacists to demonstrate their expertise and provide consultation on the use of medical narcotics in home palliative care [19]. Therefore, pharmacists in home healthcare in medically underpopulated areas should also work with attending physicians to optimize drug treatment, including the adjustment of leftover drugs and erroneous patient medication use. Moreover, it is important for pharmacists to actively participate in pre-discharge conferences to demonstrate the need and usefulness of pharmacists in collaboration with physicians [20]. Therefore, visiting pharmacists should utilize their expertise to create a bridge of communication with physicians by checking the patient’s oral medication status and proposing a reduction in medication. Furthermore, the role of the visiting pharmacist is considered important because of the acceptance of explanations by the pharmacist for patients receiving home care [21]. Previous studies have also shown that it is important to ensure that care managers are aware of the benefits of pharmacist home visits and that care managers give users and their families a good explanation of the need for such visits [22]. To achieve this, it is considered necessary for pharmacists to actively participate in discharge conferences and service manager meetings, to communicate with care managers on a regular basis, and for pharmacists to actively conduct awareness-raising activities for care managers. It was also reported that care managers had requests for pharmacists to strengthen collaboration with physicians and to provide information on home visit management guidance [23]. In the present study, as in previous studies, there is a desire for pharmacists to visit patients at home, suggesting that there is a need to strengthen collaboration with physicians and care managers. However, a new problem related to in-hospital prescribing was identified in this study, which differs from other regions where in-hospital prescribing is rare, as there are no pharmacies in the area covered by this study and most outpatient prescriptions are in-hospital. This is assumed to be due to the typical problem of no-pharmacy towns and villages, where there is no contact point for pharmacist involvement in prescribing problems, resulting in more requests for response.

Our study examined several problems related to the management of patients’ medication at home by pharmacists involved in home care in medically underpopulated areas. Previous studies have reported that 40–60% of patients with heart failure receiving home care are nonadherent [24]. Medication discontinuation triggers exacerbation of symptoms, and adherence to medication is the key to heart failure treatment, which is relevant not only in depopulated areas but also in home healthcare in urban areas. Focusing on the differences from areas with pharmacies, in the depopulated areas studied in this study, most outpatient prescriptions are in-hospital prescriptions, and out-of-hospital prescriptions are rare. This exposes a typical prescribing problem in pharmacy-free areas, where there is no contact point for pharmacists to get involved, resulting in more requests for pharmacy pharmacists to respond. As a result, they also face the problem of insecurity in managing patient compliance with medication. Medication compliance tends to decline in home healthcare because of the difficulty in establishing a support system to ensure medication compliance among healthcare professionals [16, 25]. Therefore, it is important to explore each problem individually, while perceiving the entire living environment of each patient.

Visiting pharmacists are required to check patients’ medication status through frequent visits. Unfortunately, our study revealed that the number of visits by pharmacists in medically underserved areas was so low, such as less than once a week, that pharmacists did not provide adequate medication management. In Japan, visiting nurses, caregivers, and care managers implement medication management. More than 50% of visiting nurses involved in home care felt burdened by medication management [26]. Therefore, it was thought that the intervention of visiting pharmacists in medication management was highly significant and required. Particularly in depopulated areas, the roles required for pharmacists include managing medication and adjusting leftover drugs for older adults with dementia and those living alone, collaborating with other professionals, acting as a bridge to physicians, checking medication status through frequent visits, and adhering to oral medication instructions.

It may also be important to provide careful guidance and support for medication, including resolving questions related to specialist knowledge of medicines that cannot be addressed by other professionals. In addition, there are common aspects of the current situation regarding home healthcare, although they may differ in degree regarding the lack of manpower in pharmacies and the professionalism of pharmacy pharmacists who are not mature [27]. However, in a small community such as the one in this study, the problem of care managers’ lack of understanding of pharmacist services may be resolved, since it is possible to provide adequate information to other professions, including care managers. Similarly, the differences in response to home health care services by pharmacists, frequently reported in other studies, are unlikely to have occurred in this study because this study was conducted in a pharmacy-free area and there were fewer pharmacies and pharmacists involved. The Ministry of Health, Labour and Welfare in Japan is promoting the establishment of a comprehensive community care system by 2025 with the aim of preserving the older adult’s dignity and supporting independent living so that they can continue to live their own lives in their own familiar neighborhoods as long as possible. It is based on local autonomy and initiative, and needs to be developed in accordance with local characteristics [28]. For this purpose, our results indicate that collaboration with public authorities such as the government is necessary.

This study has some limitations. Owing to interviews with care managers in certain regions, the findings might not necessarily apply to other regions. Second, even if qualitative research is generally conducted carefully, it could not be denied that the results may contain bias due to the researcher’s subjectivity and values. Third, the results of our study, which were derived from qualitative research based on current problems and the local environment, may not directly lead to generalizable findings that require a high degree of objectivity and logic, although our results may provide suggestions for solving real-world problems. Fourth, although all of the subjects in this study had caregiver background, there may be differences in the participants’ perceptions of pharmacists and the way of collaboration with pharmacists depending on their backgrounds [4]. Particularly if the care managers have a medical background, their knowledge of the medical field and understanding of the pharmacist’s competence may facilitate effective use of the pharmacist’s expertise. Fifth, this study reviewed only the perspective of care managers and did not examine the perspectives of home nurses, visiting physicians and pharmacists who are particularly involved in home healthcare, which may need to be considered in the future. Sixth, conclusions based on involvement with a small number of pharmacies may compromise objectivity.In the future, we believe that these weaknesses could be compensated for through the development of research, including a questionnaire based on the categories and concepts extracted by our study and through a series of quantitative survey studies, making it possible to propose statistically credible and real-world solutions to the factors promoting home care by pharmacists in medically underpopulated areas. At the same time, the study only worked with pharmacists in just one care-providing facility, so facility specificity cannot be ruled out. Future studies in similar locations with fewer pharmacists need to be verified to see if similar results can be obtained.

## Conclusions

It is important to first ensure the number of visits by pharmacists and collaborate with attending physicians, visiting nurses, and care managers to manage the medication of patients with dementia and older people living alone. Although there is a geographical problem, it is important for pharmacists to actively intervene in home healthcare in medically underpopulated areas, thereby promoting the understanding of pharmacists among other professionals. Community healthcare specialists and those involved in the healthcare planning system can also utilize these findings while planning home healthcare to those who live in medically underpopulated areas in Japan.

## Data Availability

The data supporting the findings of this study are available from the corresponding author upon reasonable request.

## Abbreviations

GTA: Grounded theory approach
